# Difference in Outcomes between First-Operated vs. Fellow-Operated Eyes in Patients Undergoing Bilateral Trabeculectomies

**DOI:** 10.1371/journal.pone.0136869

**Published:** 2015-08-28

**Authors:** Younhea Jung, Hae-Young L. Park, Na Young Lee, Young-Sik Yoo, Chan Kee Park

**Affiliations:** 1 Department of Ophthalmology and Visual Science, College of Medicine, Seoul St. Mary's Hospital, The Catholic University of Korea, Seoul, Korea; 2 Department of Ophthalmology and Visual Science, College of Medicine, Incheon St. Mary’s Hospital, The Catholic University of Korea, Incheon, Korea; 3 Department of Convergence Medical Science, College of Medicine, The Catholic University of Korea, Seoul, Korea; Medical University Graz, AUSTRIA

## Abstract

**Main Objective:**

To compare the course and outcome of first- and fellow-operated eyes in patients who underwent bilateral trabeculectomies and to investigate the factors associated with the difference.

**Methods:**

Preoperative characteristics, including the interval between surgeries, were compared between the first- and fellow-operated eyes in 42 patients who underwent bilateral trabeculectomies. Postoperative intraocular pressure and bleb vascularity, using postoperative anterior segment photos, were compared at various time points between the first- and fellow-operated eyes. Surgical success was evaluated at 1 year after surgery and at the final follow-up. Factors affecting the difference between the first and fellow eyes were analyzed.

**Results:**

There was no significant difference in success or failure rates at 1 year postoperatively and at the final follow-up between the first- and fellow-operated eyes. Early postoperative IOP and the degree of bleb vascularity were higher in the fellow-operated eyes (*P* = 0.001 and 0.003, respectively at week 1 postoperative). The difference in IOP between the first- and fellow-operated eyes was greater in patients whose interval between surgeries was shorter than 3 weeks (*P* = 0.026).

**Conclusions:**

In patients undergoing bilateral trabeculectomies, early postoperative IOP was higher in the fellow-operated eyes than the first-operated eyes; the difference was greater when the interval between surgeries was shorter. The first-operated eye may influence the early postoperative inflammatory response in the fellow-operated eye. Our findings have clinical implications for planning treatment of patients who may need bilateral surgery.

## Introduction

Glaucoma is a generally bilateral, but not always symmetrical, disease characterized by progressive optic neuropathy.[1,2] The mainstay of glaucoma treatment is to lower the intraocular pressure (IOP), and glaucoma filtration surgery is the most effective method of achieving that goal.[3,4] Hence, many patients need bilateral surgeries, and, in most cases, the surgeon will perform surgery on the eye with greater vision loss and more advanced glaucoma first.[5] Consequently, the first-operated eye may be more likely to have a more advanced visual field, more topical medications, and higher IOP, all of which have been reported to be risk factors for surgical failure.[6,7]

Thus, it could reasonably be hypothesized that the risk for surgical failure is greater in the first-operated eye. However, to our knowledge, no reported study has described such an outcome. Instead, some clinical experts in the glaucoma field have suggested that no matter which eye was operated on first, the fellow-operated eye may have a higher risk of postoperative complications.[8]

According to a previous study that compared the outcomes of primary trabeculectomies in bilateral eyes, the outcomes of the first and fellow eyes differed somewhat; the fellow eyes had a greater risk of Tenon’s capsule cyst formation.[8] This may indirectly indicate that the wound healing process after glaucoma surgery in the fellow-operated eye could be affected by the previous surgery in the first-operated eye. Although more investigation is needed, there is a report that the wound healing process is affected by previous surgery of the opposite eye after corneal refractive surgery. Rask et al. found a significantly faster healing cornea in the second eye than the first in patients who underwent photorefractive keratectomy.[9] Another study suggested that the corneal abrasion of the first eye affected the healing process of the corneal epithelium of the fellow eye by increasing cell proliferation.[10]

If the wound healing process is affected by the opposite eye in bilateral surgeries, there could be differences in the clinical course and outcomes in patients undergoing bilateral glaucoma surgeries. A possible mechanism for such as effect was demonstrated in a study in which systemic effects of a local wound were described. Leukocytes and systemic proinflammatory cytokines are elevated in the serum after colon resection.[11] Likewise, an ocular surgery, although localized, could cause a systemic immune and inflammatory response, which could subsequently influence the course and outcome of the fellow-eye surgery.

Considering the possible effect of the first-operated eye on wound healing in the fellow-operated eye via a systemic response, we hypothesized that wound healing in the first eye could have a systemic effect on the inflammatory process in the fellow-operated eye. The purpose of this study was to compare the course and outcomes of first- and fellow-operated eyes in patients who underwent trabeculectomies in both eyes and to investigate the factors that account for the difference.

## Materials and Methods

This retrospective chart review was approved by the Catholic University of Korea Institutional Review Board, Seoul, Korea. The institutional review board waived the need for a written consent from the participants, because of the retrospective design. Patient information was anonymized and de-identified prior to analysis. The study design followed the tenets of the Declaration of Helsinki for biomedical research.

Patients who had undergone bilateral trabeculectomy with mitomycin C at Seoul St. Mary’s Hospital, College of Medicine, Catholic University of Korea, Seoul, Korea, between January 2005 and February 2014 were enrolled. All patients underwent trabeculectomies in both eyes, all performed by one expert surgeon (CKP). We excluded patients who had undergone previous surgical procedures that involved conjunctiva manipulation to reduce its possible influence on the outcome of glaucoma surgeries. Patients with less than 1 year of follow-up were also excluded.

Preoperative data were gathered from reviewing patient charts, which included presence of systemic diseases such as diabetes mellitus or hypertension, acquired by history taking, type of glaucoma, time interval between first and fellow surgery, best corrected visual acuity (logMAR), mean deviation on a Humphrey visual field analyzer (24–2 Swedish Interactive Threshold Algorithm Standard) obtained before surgery, IOP measured by Goldmann applanation tonometry on the day before surgery, and type and duration of antiglaucomatous medications.

Glaucoma surgeries were performed under general or retrobulbar anesthesia. Postoperative follow-up occurred at 1 and 2 days; 1, 2, and 3 weeks; 1, 3, and 6 months; and 1 year after surgery. At each follow-up, visual acuity, IOP, number of antiglaucomatous medications, and surgery-related complications, including shallow anterior chamber, choroidal detachment, and hyphema, were recorded, and anterior segment photographs were taken. We analyzed vascularity of the bleb based on anterior segment photographs using the Moorfields Bleb Appearance Grading System.5[12] The examiner (YJ) was blinded to the information related to the photographs including which eye was operated first.

The success rate was judged using common definitions.[13] An IOP ≤ 21 mmHg with no antiglaucomatous medication was deemed a complete success, and an IOP ≤ 21 mmHg with antiglaucomatous medications was classified as a qualified success. A surgical failure was defined as an IOP > 21 mmHg with medication(s), progressive glaucomatous visual field loss, combined with an increase in cupping of the optic disc regardless of the IOP, or the need for further antiglaucomatous surgical procedures.

Statistical analyses were performed using the SPSS software (ver. 17.0; SPSS Inc., Chicago, IL). Characteristics between the first and fellow-operated eye were compared with Student’s *t*-test for continuous parameters and the χ^2^ test for categorical parameters. Mann-Whitney test was used for nonparametric data. Repeated-measures ANOVA was used to compare the pattern of IOP change between the first- and fellow-operated eyes. To identify factors associated with the difference between first and fellow eyes, univariate and multivariate regression analyses were performed. *P* values < 0.05 were considered to indicate statistical significance.

## Results

**Fig 1:**
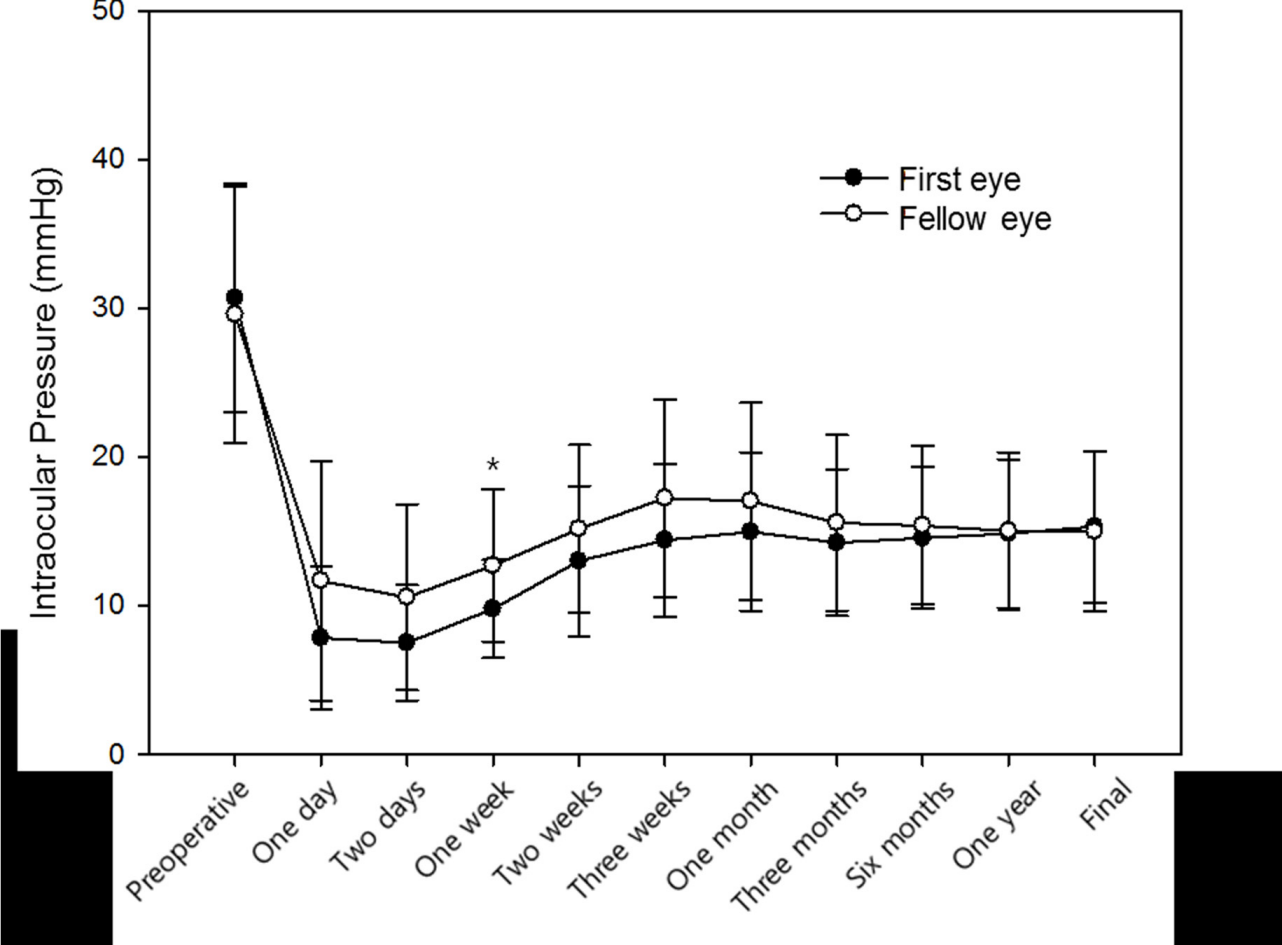
Comparison of preoperative and postoperative intraocular pressure in the first- and fellow-operated eyes. (*P* = 0.023; repeated measures analysis of variance). * Statistically significant difference between groups.

In total, we identified 63 patients who had bilateral trabeculectomies at our institution between January 2005 and February 2014. After excluding five patients due to short follow-up periods and 16 patients due to previous ocular surgeries involving the conjunctiva, 42 patients were enrolled. Patient demographics are shown in Table 1. All patients had undergone surgery due to the same diagnosis on both eyes. Postoperative needling or 5-FU injections were not performed in any case. Characteristics of first-operated eyes and fellow-operated eyes are compared in Table 2. Age at the time of surgery and preoperative and postoperative visual acuity were similar between the first- and fellow-operated eyes (*P* = 0.861, 0.595, and 0.861, respectively). The mean follow-up period was significantly longer for the first-operated eyes (26.71 ± 14.69 months) than the fellow-operated eyes (20.30 ±13.19 months; *P* = 0.039). Mean deviation on the Humphrey visual field test was significantly worse in the first-operated eyes (-15.89 ±10.23 dB) than the fellow-operated eyes (-10.95 ± 10.07 dB; *P* = 0.029). Preoperative number of antiglaucomatous medications was not statistically different between first-(3.12 ± 0.50) and fellow-operated eyes (3.02 ± 0.52, *P* = 0.396, Table 2). The type of antiglaucomatous medications was also not statistically different (*P* = 0.846, S1 Fig). The duration of preoperative antiglaucomatous medications was significantly longer in the fellow-operated eyes (90.48 ± 60.97 months) than the first-operated eyes (84.02 ± 60.59 months, *P* = 0.047). Postoperative IOP was significantly higher in the fellow-operated eyes at postoperative day 1 and 2 and weeks 1, 2, and 3 (*P* = 0.010, 0.008, 0.003, 0.035, and 0.032, respectively). The pattern of postoperative IOP change differed between the two groups (*P* = 0.023), and using a Bonferroni-adjusted significance level of 0.004, there were significant differences in IOP at postoperative week 1 (Fig 1). The number of medications was not significantly different between the two groups preoperatively and postoperatively (Table 2).

**Table 1:** Patient Demographics.

Characteristic	No. of patients	%
Total number of patients (eyes)	42 (84)	
Gender (Male/Female)	26/16	
First-operated eye		
Right	23	54.8
Left	19	45.2
Type of glaucoma (patients)		
Primary open-angle glaucoma	36	85.7
Chronic angle closure	4	9.5
Pseudoexfoliation	1	2.3
Steroid induced	1	2.3
Time between first and fellow surgery	7.07 ± 7.73	
(months, mean ± SD (range))	(1 week—30 months)	
Diabetes mellitus	13	31
Systemic hypertension	15	35.7

**Table 2:** Characteristics of first- and fellow-operated eyes.

Characteristic	First-operated eyes	Fellow-operated eyes	P value
Age at the time of surgery (years, mean ± SD)	49.66 ± 18.60	50.38 ± 18.74	0.861
Follow up period (months, mean ± SD)	26.71 ±14.69	20.30 ±13.19	0.039*
Visual acuity (logMAR)			
Before surgery, mean ± SD	0.24 ± 0.43	0.21 ± 0.48	0.595
At final visit, mean ± SD	0.27 ± 0.47	0.26 ± 0.42	0.861
Visual field mean deviation (dB)			
Before surgery, mean ± SD	-15.89 ± 10.23	-10.95± 10.07	0.029*
Intraocular pressure (mmHg)			
Before surgery	30.69 ± 7.64	29.57 ± 8.63	0.531
Day 1	7.83 ± 4.81	11.66 ± 8.04	0.010*
Day 2	7.50 ± 3.87	10.57± 6.26	0.008*
Week 1	9.78 ± 3.30	12.69 ± 5.11	0.003*
Week 2	13.00 ± 5.05	15.16 ± 5.63	0.035*
Week 3	14.40 ± 5.17	17.23 ± 6.67	0.032*
1 Month	14.97 ± 5.33	17.02 ± 6.64	0.131
3 Months	14.23 ± 4.94	15.57 ± 5.93	0.267
6 Months	14.54 ± 4.76	15.38 ± 5.33	0.452
1 Year	14.85 ± 5.00	15.02 ± 5.30	0.788
At final visit	15.30 ± 5.12	15.00 ± 5.36	0.422
Number of medications			
Before surgery	3.12 ± 0.50	3.02 ± 0.52	0.396
Day 1	0	0	
Day 2	0	0	
Week 1	0	0	
Week 2	0.02 ± 0.15	0	0.317
Week 3	0.04 ± 0.21	0.09 ± 0.29	0.400
1 Month	0.09 ± 0.29	0.21 ± 0.47	0.205
3 Months	0.28 ± 0.55	0.45 ± 0.70	0.236
6 Months	0.30 ± 0.64	0.50 ± 0.77	0.206
1 Year	0.50 ± 0.86	0.66 ± 0.95	0.367
At final visit	0.78 ± 1.04	0.92 ± 1.04	0.416

* Statistically significant value.

**Fig 2:**
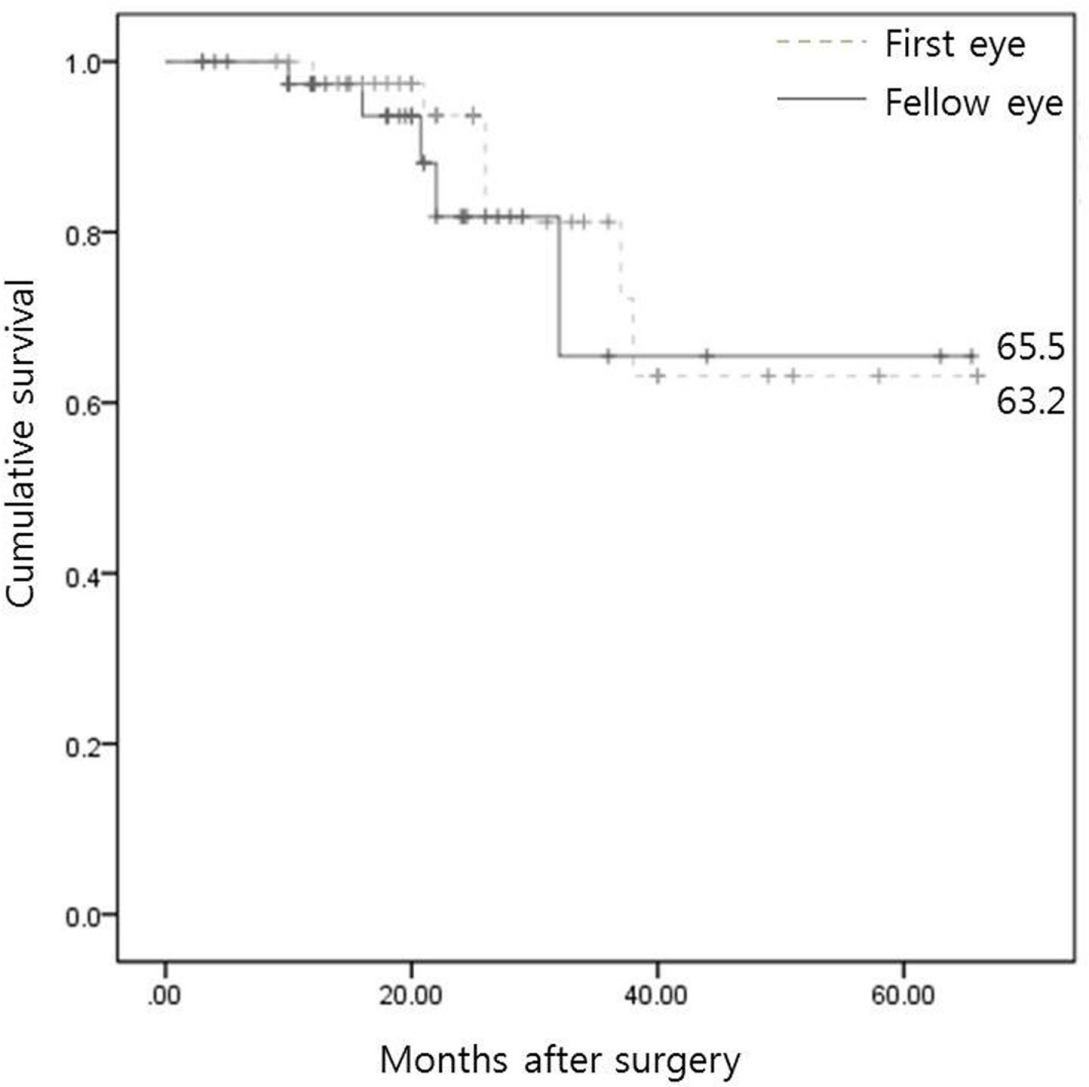
Kaplan-Meier analysis for surgical success during the follow-up period in first- and fellow-operated eyes. The curves were not significantly different between the two groups (*P* = 0.564; log-rank test).

Percentages of complete success at postoperative 1 year and at the final visit were higher in the first group (69.0% and 47.6%) than the fellow group (59.5% and 42.9%), but the ratio of complete success, qualified success, or failure was not significantly different between the two groups (*P* = 0.292 and 0.797, respectively; Tables 3 and 4). There was no difference in the survival curves during the follow-up period between the groups (P = 0.564; Fig 2). There was no significant difference in the number of postoperative complications, including choroidal detachment, shallow anterior chamber, and hyphema, in first and fellow eyes (P = 1.000, 0.974, and 1.000, respectively; Table 5).

**Table 3:** Success rate of first- and fellow-operated eyes at postoperative year 1.

Postoperative year 1	Complete success	Qualified success	Failure	P value
First-operated eyes	29 (69.0%)	9 (21.4%)	4 (9.5%)	0.292
Fellow-operated eyes	25 (59.5%)	15 (35.7%)	2 (4.8%)	

**Table 4:** Success rate of first- and fellow-operated eyes at the final visit.

At final visit	Complete success	Qualified success	Failure	P value
First-operated eyes	20 (47.6%)	16 (38.1%)	6 (14.3%)	0.797
Fellow-operated eyes	18 (42.9%)	19 (45.2%)	5 (11.9%)	

**Table 5:** Postoperative complications in first- and fellow-operated eyes.

	First-operated eyes	Fellow-operated eyes	P value
Choroidal detachment	3 (7.1%)	3 (7.1%)	1.000
Shallow anterior chamber	5 (11.9%)	6 (14.3%)	0.974
Hyphema	1 (2.4%)	1 (2.4%)	1.000

**Fig 3:**
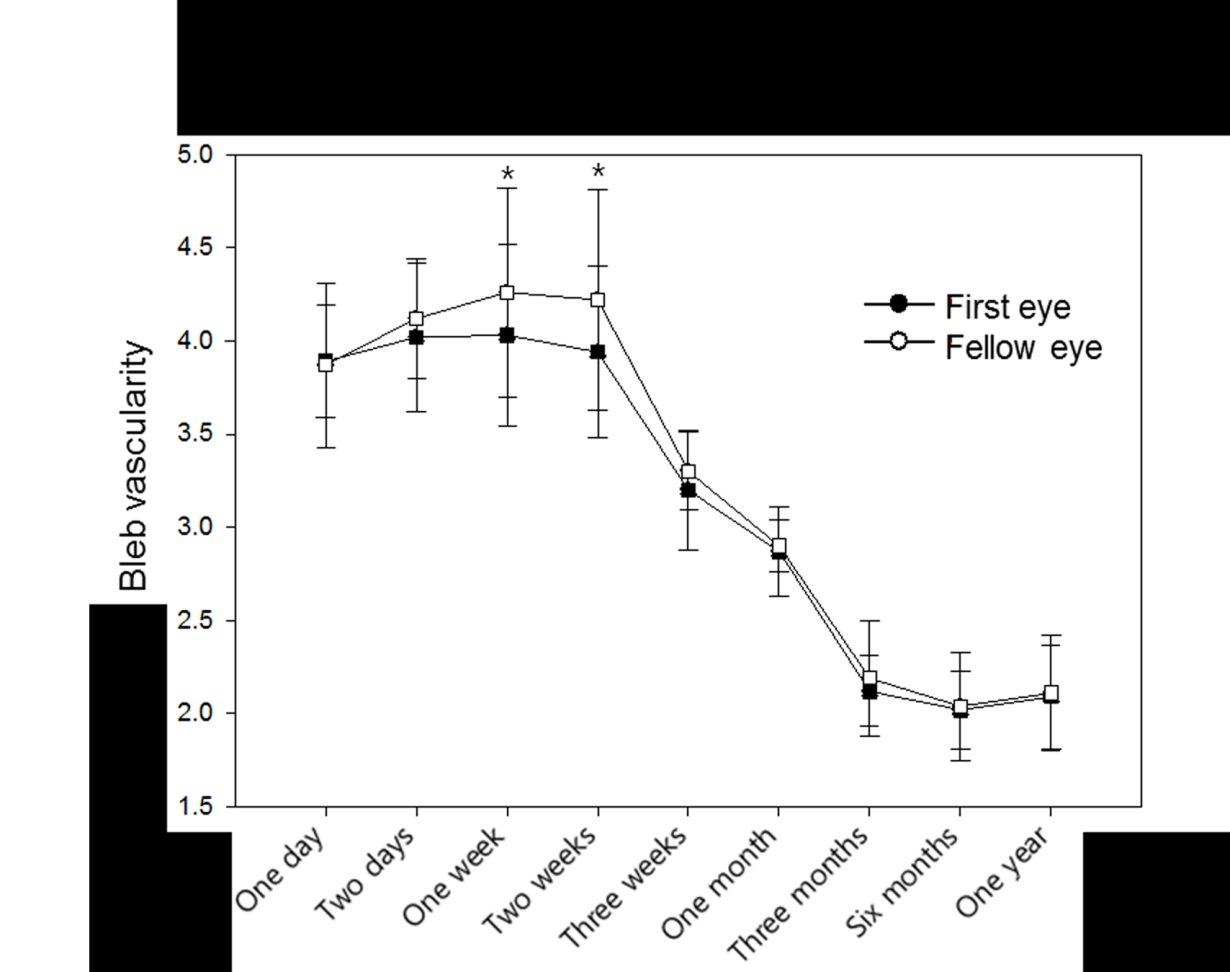
Comparison of bleb vascularity between first- and fellow- operated eyes. The degree of vascularity was significantly higher in the fellow-operated eyes at postoperative weeks 1 and 2 (*P* = 0.003 and 0.005, respectively). The examiner was blinded to the information related to the photographs, including which eye was operated first, that were used to grade bleb vascularity at all time points.

**Fig 4:**
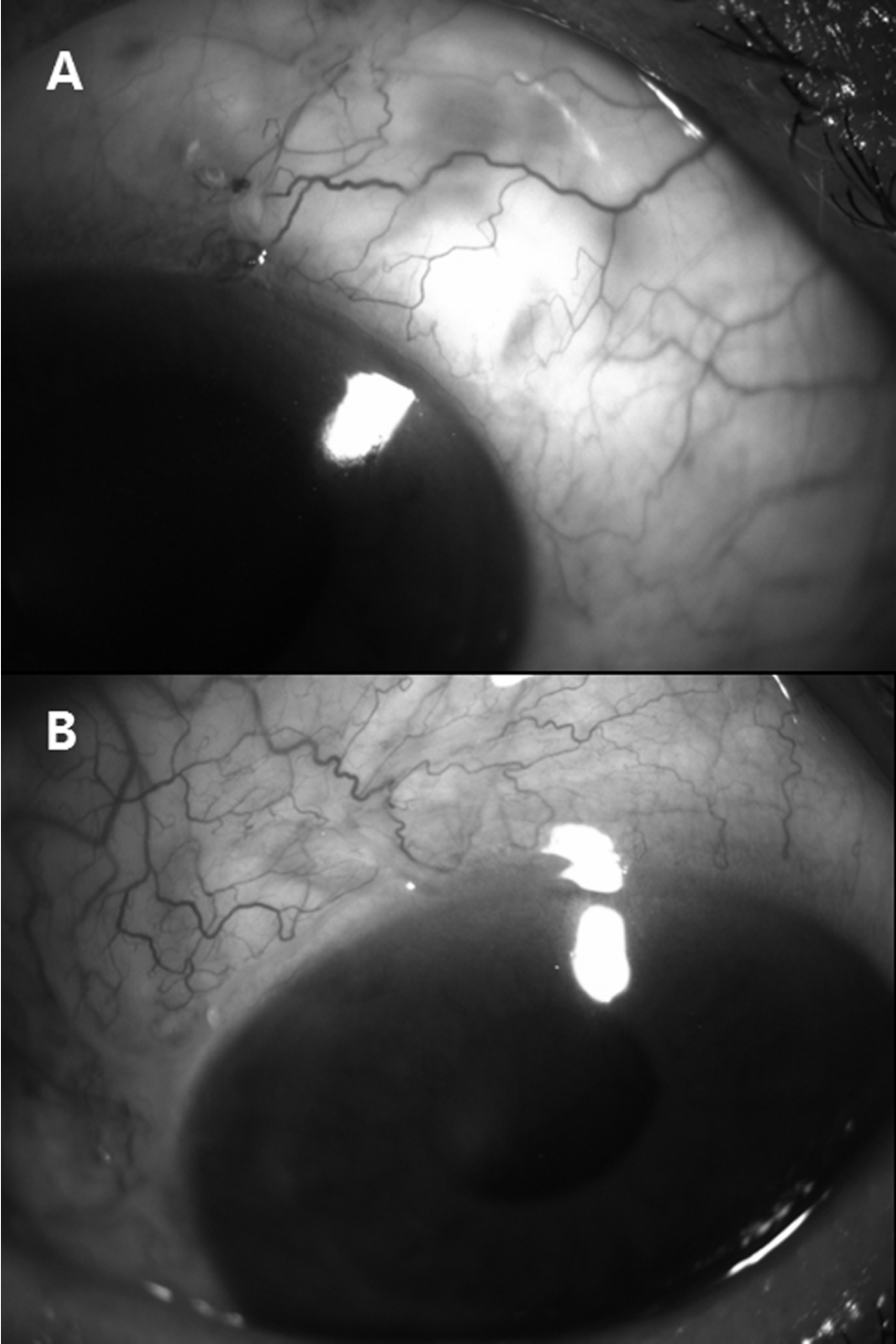
Representative photographs of bleb vascularity at postoperative week 1. Vascularity was more severe in the fellow-operated eye (B) than in the first-operated eye (A). The interval between these surgeries was 2 weeks.

The degree of bleb vascularity was significantly higher in the fellow-operated eyes at postoperative weeks 1 and 2 (*P* = 0.003 and 0.005, respectively; Fig 3). Fig 4 shows representative images of bleb vascularity in first and fellow eyes.

**Fig 5:**
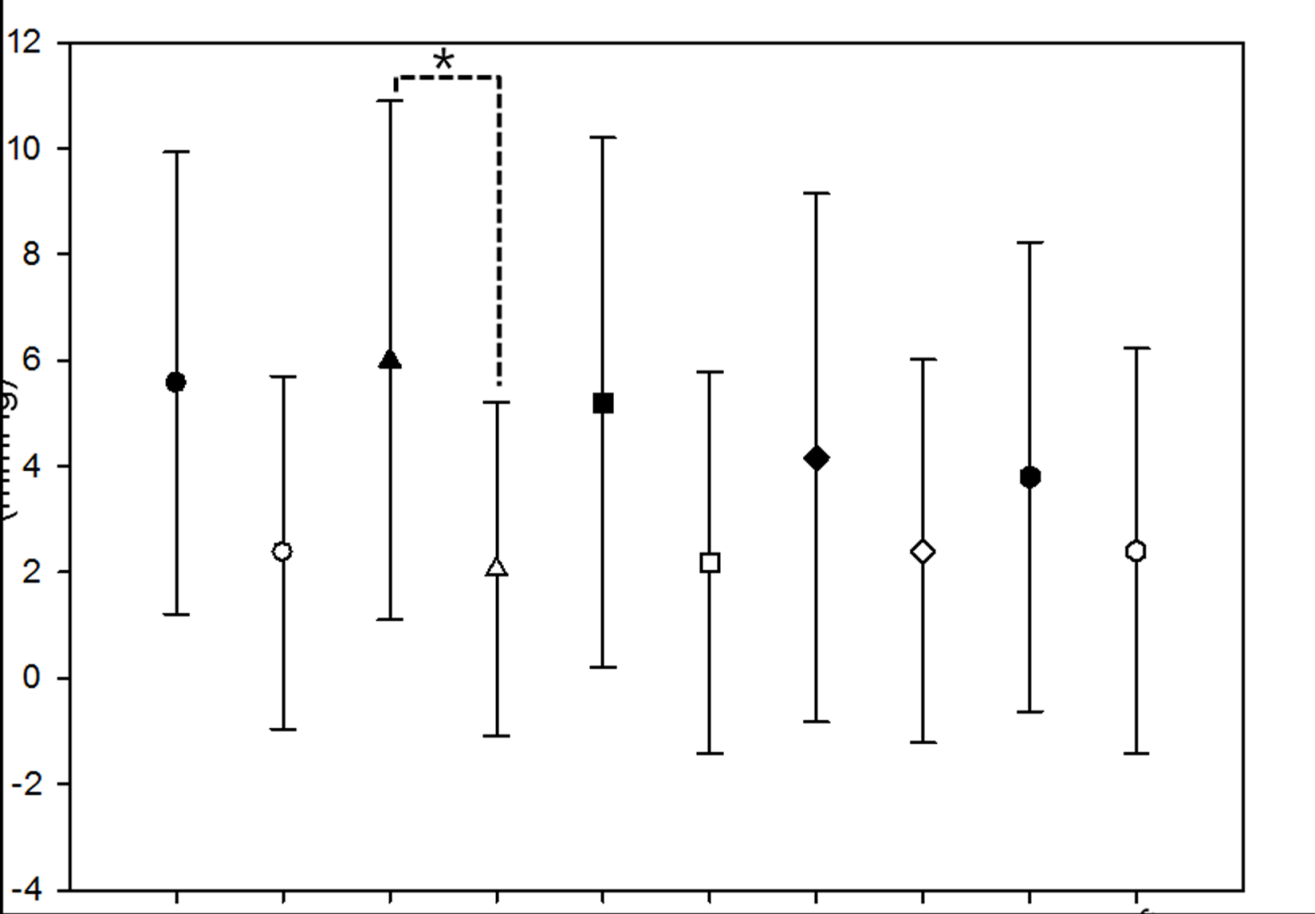
Difference in IOP between the first- and fellow-operated eyes according to time interval between surgeries. The IOP difference of postoperative week 1 was significantly greater in patients with a shorter interval between the surgery on the first and fellow eyes. This difference was significantly greater when the interval was less than 3 weeks compared to when it was greater than 3 weeks.

To determine factors associated with the difference between first and fellow eyes, a linear regression analysis was performed. The difference in IOP between first and fellow eyes was greatest at postoperative week 1; thus, this was used as the dependent variable in the regression analysis (Table 2). The interval between the first and fellow eye surgeries did not show a linear relationship with the difference in IOP between first and fellow eyes (*P* = 0.106, data not shown). However, the difference in IOP between the first and fellow eyes was greater in patients where the interval between the first and fellow eyes was shorter; the difference was significantly greater when the interval was less than 3 weeks (*P* = 0.041, Fig 5). Age, gender, diabetes, and hypertension were not statistically significantly associated with the difference in IOP between first and fellow eyes. In a multivariate analysis, the interval between first and fellow eye surgeries was associated with the IOP difference between first and fellow eyes at postoperative week 1; when the interval was shorter than 3 weeks, the difference in IOP was greater (*P* = 0.028; Table 6).

**Table 6:** Multivariate analysis of the difference between first- and fellow-operated eyes.

Difference in intraocular pressure at postoperative week 1	Regression coefficient	P value
Age	0.099	0.065
Gender	-0.089	0.585
Diabetes Mellitus	-0.102	0.536
Hypertension	-0.129	0.455
Three-week interval between surgeries	-5.436	0.028*

* Statistically significant value.

## Discussion

The aim of the present study was to compare the outcomes of first- and fellow-operated eyes in patients who underwent bilateral glaucoma surgeries and to examine factors related to the difference. The success rates of the first- and fellow-operated eyes were similar. However, the preoperative characteristics of the first- and fellow-operated eyes differed; the first-operated eyes had significantly worse mean deviation and, although not statistically significant, slightly higher preoperative IOP than fellow-operated eyes. Also, although not statistically significant, age at the time of surgery was higher in the fellow-operated eyes. Previous studies have reported glaucoma severity, as measured by mean deviation on visual field tests [6], higher preoperative IOP [14,15], and younger age [14,16] as risk factors for failure of glaucoma surgeries. Despite the worse mean deviation of the first-operated eyes, the success rates of first and fellow eyes were similar. This may be an indirect indication that the fellow-operated eyes, in fact, have a higher failure rate.

The present study also showed that early postoperative IOP was higher and bleb vascularity was more severe in the fellow-operated eyes. The difference in IOP was the greatest at postoperative week 1 and was statistically significant until postoperative week 3. This difference was greater in patients with a shorter time interval between first and fellow eye surgeries.

Early postoperative IOP has been related with the probability of surgical success. Okimoto et al.[17] showed that patients whose IOP was less than 8 mmHg at 2 weeks after trabeculectomy demonstrated better IOP control and higher rate of success. Won and Sung [18] reported that hypertensive phase after Ahmed glaucoma valve implantation was related with lower rate of complete success. Our results showed fellow-operated eyes had higher postoperative IOP which may be related with lower success rate based on these previous studies. However, the success rate between first- and fellow-operated eyes was similar, perhaps this may be due to worse mean deviation of the first-operated eyes as mentioned earlier.

In our study, the duration of glaucoma medications prior to surgery was longer in fellow-operated eyes. Longer duration of medication has been associated with higher rate of trabeculectomy failure due to subclinical inflammatory changes caused by exposure to benzalkonium chloride.[19] This may have played a role in the higher IOP of the fellow-operated eyes in the early postoperative period. However, in our study, the patients with a longer time interval between first- and fellow-operated eyes, with possibly more subclinical inflammatory changes in the fellow-operated eye, showed less IOP difference than did those with a shorter time interval.

The mechanism underlying this finding remains unclear. It may be the result of other potential confounding factors unrecorded in the charts that affected the order of surgery or other patient-related factors such as postoperative withdrawal of eyedrops in the fellow eye. However, patients with glaucoma in the fellow eye are instructed preoperatively and postoperatively to continue their medications. Another hypothesis for higher IOP and more severe vascularity in the fellow-operated eyes is that the wound healing in the first-operated eye triggered a systemic mechanism, which in turn affects wound healing, shown as higher IOP and more severe bleb vascularity, in the fellow eye. This effect of the first-operated eye may decrease eventually with time, which may account for the smaller IOP difference between first and fellow eyes in patients with longer interval between the surgeries. However, this is our speculation, and more research is warranted to account for the effect of first-operated eye on the fellow-operated eye. Other studies have studied the effect of first-operated eyes on the fellow-operated eyes. Mietz et al.[8] compared the results of primary trabeculectomies of bilateral eyes and reported that fellow eyes had an increased risk of Tenon’s capsule cysts. They also proposed that the surgery in the first eye activated a mechanism that influenced wound healing in the fellow eye. Estil et al. also showed that a previous corneal erosion in the fellow eye resulted in faster healing of the second eye, and explained that this corneal healing process was influenced by systemic factor(s).[10] Our data are consistent with those studies and further demonstrated that this systemic effect may influence the early phase of the fellow eye’s wound healing, which corresponds to the inflammatory phase.[20]

Regarding the time interval between the first and fellow eye surgeries, Rask et al. reported that the corneal epithelium of the second eye healed faster than the first eye in patients subjected to photorefractive keratectomy, with a 1-week interval.[9] Patients with longer intervals did not show a significant difference. In the present study, the difference in IOP between first and fellow eyes was greater in patients with a less than 3-week interval than in patients with longer intervals between surgeries. Although the specific time interval may differ due to the different wound healing mechanisms between the corneal epithelium and the conjunctiva, both studies demonstrate that the systemic effect of the first eye on the fellow eye decreases eventually.

Our study had some limitations due to its retrospective design. We could not adjust or account for all potential confounders which may have affected first- and fellow-operated eyes differently. The use of antiglaucomatous medications prior to the visit to our hospital was based on medical records and referral letters the patients were required to bring with them on their initial visit, of which the reliability may be inconsistent amongst patients. Nevertheless, it could reasonably be assumed that the reliability of information within a patient, between first- and fellow-operated eyes, may be less inconsistent. Another limitation is that the surgeon had knowledge of the first-operated eye, which may have affected the surgical technique of the fellow-eye surgery. However, early postoperative complications, including choroidal detachment and shallow anterior chamber, were similar between the first- and fellow-operated eyes. In addition, not only postoperative IOP, but also vascularity, which is more difficult to control by surgical techniques, was different between first- and fellow-operated eyes.

In conclusion, the results of this study suggest that in patients subjected to bilateral glaucoma surgeries, the early postoperative IOP is higher in the fellow-operated eyes than the first-operated eyes. This difference in IOP was greater in patients with shorter intervals between first and fellow surgeries. Surgeons should be more careful of early IOP increases in fellow eyes, and when possible allow some time between first and fellow surgeries, which we recommend to be more than 3 weeks based on this study.

## Supporting Information

S1 Fig Preoperative medications of first-operated eyes and fellow-operated eyes. (*P* = 0.846, Fisher’s Exact Test).
(TIF)
