# Supplementary figures and images for: Difference in Outcomes between First-Operated vs. Fellow-Operated Eyes in Patients Undergoing Bilateral Trabeculectomies
Source: PLoS One. 2015 Aug 28;10(8):e0136869. doi: 10.1371/journal.pone.0136869 (PMC4552850; doi:10.1371/journal.pone.0136869)

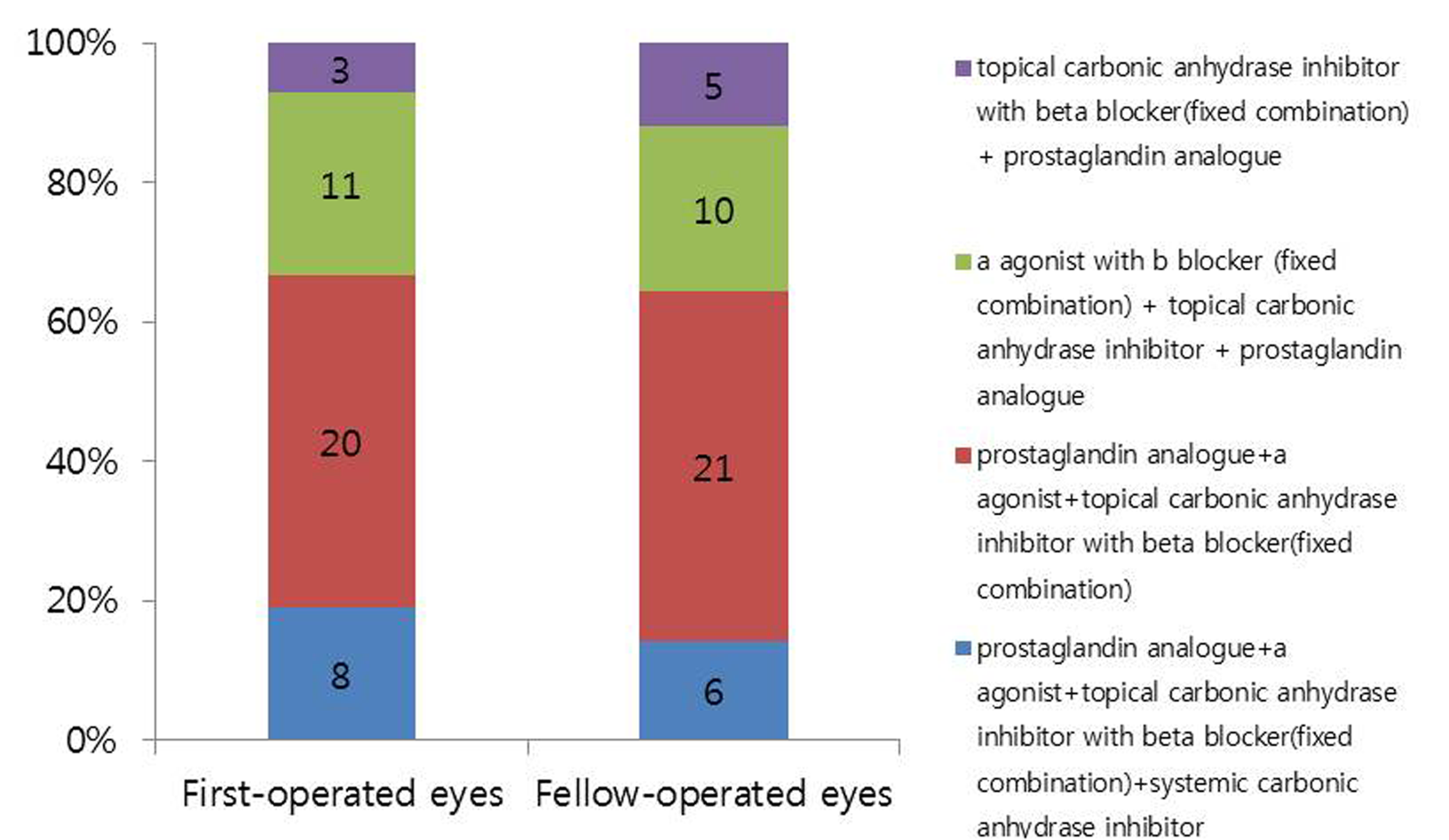

Supplement: S1 Fig — (P = 0.846, Fisher’s Exact Test). (TIF) [file pone.0136869.s001.tif]
